# Cu-doped ZnO nanorod arrays: the effects of copper precursor and concentration

**DOI:** 10.1186/1556-276X-9-199

**Published:** 2014-05-01

**Authors:** Musbah Babikier, Dunbo Wang, Jinzhong Wang, Qian Li, Jianming Sun, Yuan Yan, Qingjiang Yu, Shujie Jiao

**Affiliations:** 1 Department of Opto-Electric Information Science, School of Materials Science and Engineering, Harbin Institute of Technology, Harbin 150001, China

**Keywords:** Zinc oxide, Nanostructures, Doping, Hydrothermal crystal growth, Photoluminescence

## Abstract

Cu-doped ZnO nanorods have been grown at 90°C for 90 min onto a quartz substrate pre-coated with a ZnO seed layer using a hydrothermal method. The influence of copper (Cu) precursor and concentration on the structural, morphological, and optical properties of ZnO nanorods was investigated. X-ray diffraction analysis revealed that the nanorods grown are highly crystalline with a hexagonal wurtzite crystal structure grown along the *c*-axis. The lattice strain is found to be compressive for all samples, where a minimum compressive strain of −0.114% was obtained when 1 at.% Cu was added from Cu(NO_3_)_2_. Scanning electron microscopy was used to investigate morphologies and the diameters of the grown nanorods. The morphological properties of the Cu-doped ZnO nanorods were influenced significantly by the presence of Cu impurities. Near-band edge (NBE) and a broad blue-green emission bands at around 378 and 545 nm, respectively, were observed in the photoluminescence spectra for all samples. The transmittance characteristics showed a slight increase in the visible range, where the total transmittance increased from approximately 80% for the nanorods doped with Cu(CH_3_COO)_2_ to approximately 90% for the nanorods that were doped with Cu(NO_3_)_2_.

## Background

ZnO semiconductor attracted considerable research attention in the last decades due to its excellent properties in a wide range of applications. ZnO is inherently an n-type semiconductor and has a wide bandgap of approximately 3.37 eV and a large exciton binding energy of approximately 60 meV at room temperature. As mentioned above, ZnO is a promising semiconductor for various applications such as UV emitters and photodetectors, light-emitting diodes (LEDs), gas sensors, field-effect transistors, and solar cells [1-6]. Additionally, ZnO resists radiation, and hence, it is a suitable semiconductor for space technology applications. Recently, ZnO nanostructures have been used to produce short-wavelength optoelectronic devices due to their ideal optoelectronic, physical, and chemical properties that arise from a high surface-to-volume ratio and quantum confinement effect [6-8]. Among the ZnO nanostructures, ZnO nanorods showed excellent properties in different applications and acted as a main component for various nanodevices [1,2,9-11]. Previous research showed that the optical and structural properties of ZnO nanorods can be modified by doping with a suitable element to meet pre-determined needs [12,13]. The most commonly investigated metallic dopants are Cu and Al [13-15]. Specifically, copper is known as a prominent luminescence activator, which can enhance the green luminescence band by creating localized states in the bandgap of ZnO [16-19]. Previous research showed that Cu has high ionization energy and low formation energy, which speedup the incorporation of Cu into the ZnO lattice [16,20]. Experimentally, it was observed that the addition of Cu into ZnO-based systems has led to the appearance of two defective states at +0.45 eV (above the valence band maximum) and −0.17 eV (below the conduction band minimum) [21,22]. Currently, a green emission band was observed for many Cu-doped ZnO nanostructures grown by different techniques [23,24]. Moreover, Cu as a dopant gained more attention due to its room-temperature ferromagnetism, deep acceptor level, some similar properties to those of Zn, gas sensitivity, and enhanced green luminescence [15-17]. However, there are several points that have to be analyzed such as the effect of the copper source on the structural, morphological, and optical properties of Cu-doped ZnO. Moreover, the luminescence and the structural properties of Cu-doped ZnO nanorods are affected by different parameters such as growth conditions, growth mechanism, post growth treatments, and Cu concentration. Despite the promising properties, research on the influence of Cu precursors on Cu-doped ZnO nanorod properties remains low.

ZnO nanostructures can be synthesized by a variety of techniques including vapor-phase transport, chemical vapor deposition, sol-gel, condensation, spray pyrolysis, and hydrothermal method. Among these methods, the hydrothermal method is used to prepare ZnO nanorods due to its low cost and simplicity [16,25,26].

In order to improve the structural and optical properties of Cu-doped ZnO nanorods, the effect of the Cu precursor is worth clarification. In the study reported here, we have synthesized pure and Cu-doped ZnO nanorods onto a quartz substrate pre-coated with a ZnO seed layer using the hydrothermal method. The main focus has been put on the effect of the copper precursor on the morphology, structural, transmittance, and photoluminescence properties of the synthesized ZnO nanorods.

## Methods

The nanorod growth was accomplished in two steps: (1) the sputtering of ZnO seed layer to achieve highly aligned Cu-doped ZnO nanorods [27] and (2) the nanorod growth using the hydrothermal method.

### Sputtering of ZnO seed layer

Prior to the nanorod growth, a 120-nm-thick seed layer of undoped ZnO was deposited onto a quartz substrate using RF magnetron sputtering at room temperature. Before the deposition of the ZnO seed layer, a surface treatment of the quartz substrate was conducted using acetone, ethanol, and deionized water for 10 min for each at RT and then dried in air. Pure ZnO (99.999%) with a 50-mm diameter and 5-mm thickness was used as the ZnO target. The seed layer sputtering was accomplished in a mixture of O and Ar gas atmosphere with the gases' flow rates of 2.5 and 35 sccm, respectively. The base pressure attained was 10^−4^ Pa, and the working pressure was 1 Pa during sputtering. The sputtering power was 100 W. In order to remove the contaminants from the ZnO target, pre-sputtering for 10 min was performed. Finally, the ZnO-sputtered seed layer thin films were annealed at 500°C for 30 min.

### Nanorod growth

Undoped and Cu-doped ZnO nanorods were grown by the hydrothermal method on a quartz substrate seeded with the ZnO thin film using hexamethylenetetramine (HMT) ((CH_2_)_6_ N_4_), zinc acetate dihydrate (Zn(CH_3_COO)_2_ · 2H_2_O), and either cupric acetate (Cu(CH_3_COO)_2_ · H_2_O) or cupric nitrate (Cu(NO_3_)_2_ · 3H_2_O) as hydroxide, zinc (Zn), and copper (Cu) precursors, respectively. The nanorod growth was accomplished by suspending the substrates in a conical flask containing the aqueous solution that was prepared from zinc acetate (0.025 M) and HMT (0.025 M). Before suspending the samples, the aqueous solution was magnetically stirred for 30 min. The flask that contains the equimolar aqueous solution was placed in a combusting waterbath deposition system at 90°C for 90 min. After the nanorods were grown, the samples were removed from the beakers, rinsed in deionized water several times to remove the unreacted materials, and then finally dried in an oven at 60°C for 2 h. In order to introduce the Cu dopants, either cupric acetate (0.025 M) or cupric nitrate (0.025 M) was added directly to the reaction path. To study the effects of Cu concentration and precursor on the Cu-doped ZnO nanorods, five samples (S1 to S5) were prepared. For simplicity, the undoped ZnO nanorod (sample S1) was used as a reference sample. Samples S2 and S3 were doped with 1 and 2 at.% of Cu, respectively, from Cu(CH_3_COO)_2_. Samples S4 and S5 were doped with 1 and 2 at.% of Cu, respectively, from Cu(NO_3_)_2_. For more details, see Table 1 to clarify the concentrations and precursors for each sample.

**Table 1:** Precursors, concentrations, and crystal parameters of undoped and Cu-doped ZnO nanorods

	**S1**	**S2**	**S3**	**S4**	**S5**
Zn precursor	Zn ACT	Zn ACT	Zn ACT	Zn ACT	Zn ACT
OH precursor	HMT	HMT	HMT	HMT	HMT
Cu precursor	-	Cu acetate	Cu acetate	Cu nitrate	Cu nitrate
Cu (at.%)	-	1	2	1	2
FWHM (degrees)	0.096	0.087	0.087	0.099	0.134
*c* (Å)	5.186	5.192	5.200	5.201	5.184

### Characterization and measurements

In order to characterize the structure of the grown nanorods, X-ray diffraction (XRD) measurements were performed using a MiniFlex-D/MAX-rb with CuKα radiation. The morphology of the hydrothermally grown nanorods was investigated by field emission scanning electron microscope (SEM) using SEM Helios Nanolab 600i (Hillsboro, OR, USA). Photoluminescence (PL) spectra were measured at room temperature with an excitation source of 325-nm wavelength using a He-Cd laser. Transmittance measurements were recorded by a UV-vis spectrophotometer (Phenix –1700 PC, Shanghai, China).

## Results and discussion

### Crystal structure

**Figure 1:**
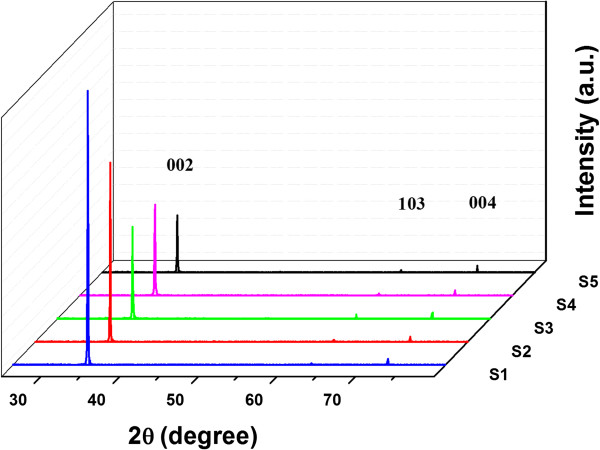
XRD patterns of undoped and Cu-doped ZnO nanorods.

Figure 1 shows the XRD patterns of the undoped and Cu-doped ZnO nanorod samples grown with varied concentrations and doped from two different Cu precursors. Clearly, a strong and narrow peak corresponding to ZnO (002) is observed, indicating that all samples possess a hexagonal wurtzite crystal structure with highly preferred growth direction along the *c*-axis perpendicular to the substrate. Additionally, there were two weak diffraction peaks observed at around 63.2° and 72.8°, which correspond to ZnO (103) and ZnO (004), respectively. For the Cu-doped ZnO nanorod samples, no other diffraction peaks are observed, only ZnO-related peaks, which is consistent with previous results [6,16,18,28]. It may be seen that the diffraction intensity from the (002) plane is more pronounced for the undoped ZnO nanorods (sample S1) and decreases with the increase of Cu concentration regardless of the Cu precursor, indicating that the incorporation of Cu dopants into the ZnO lattice induces more crystallographic defects and hence degrades the crystal quality [16,28]. In terms of Cu precursor, the samples doped with 1 and 2 at.% of Cu from Cu(CH_3_COO)_2_ (samples S2 and S3) exhibited strong diffraction intensities from the (002) plane compared to the samples doped with 1 and 2 at.% of Cu from Cu(NO_3_)_2_ (samples S4 and S5). This result suggests that the samples doped with Cu(CH_3_COO)_2_ (S2 and S3) have a low concentration of crystallographic defects. The decrease in the crystal quality of the samples doped with Cu(NO_3_)_2_ (S4 and S5) might be attributed to (i) the formation of [Cu_Zn_-Zn_i_]^*x*^ complexes and/or (ii) the lack of hydrolysis process in ${\mathrm{NO}}_{3}^{-}$, which could increase the anion vacancies in the ZnO lattice [29,30]. However, the strong (002) peaks' positions of the Cu-doped nanorods showed a slight shift toward a lower angle relative to the undoped nanorods. This shift is more significant for sample S3. On the other hand, previous research showed that at low concentrations (<1.5 at.%) of Cu, the peak position is not significantly affected by Cu doping, while at high concentration, a slight shift towards higher angles is reported due to the substitution of Zn^2+^ (ionic radii = 0.074 nm) by Cu^2+^ (ionic radii = 0.057 nm) [30,31]. Additionally, these changes in crystallinity might be due to the changes in the atomic environment as a result of Cu incorporation into the ZnO lattice. It is evident that there is a slight lattice deformation in the Cu-ZnO lattice, which may be assigned to the diminishing Cu_Zn_-O bonds [32]. In this study, with up to 2% Cu concentration from the two precursors, neither the Cu nor CuO phases are observed in the XRD measurements, which indicates that the Cu impurities are dissolved completely in the ZnO crystal lattice [26,30].

**Figure 2:**
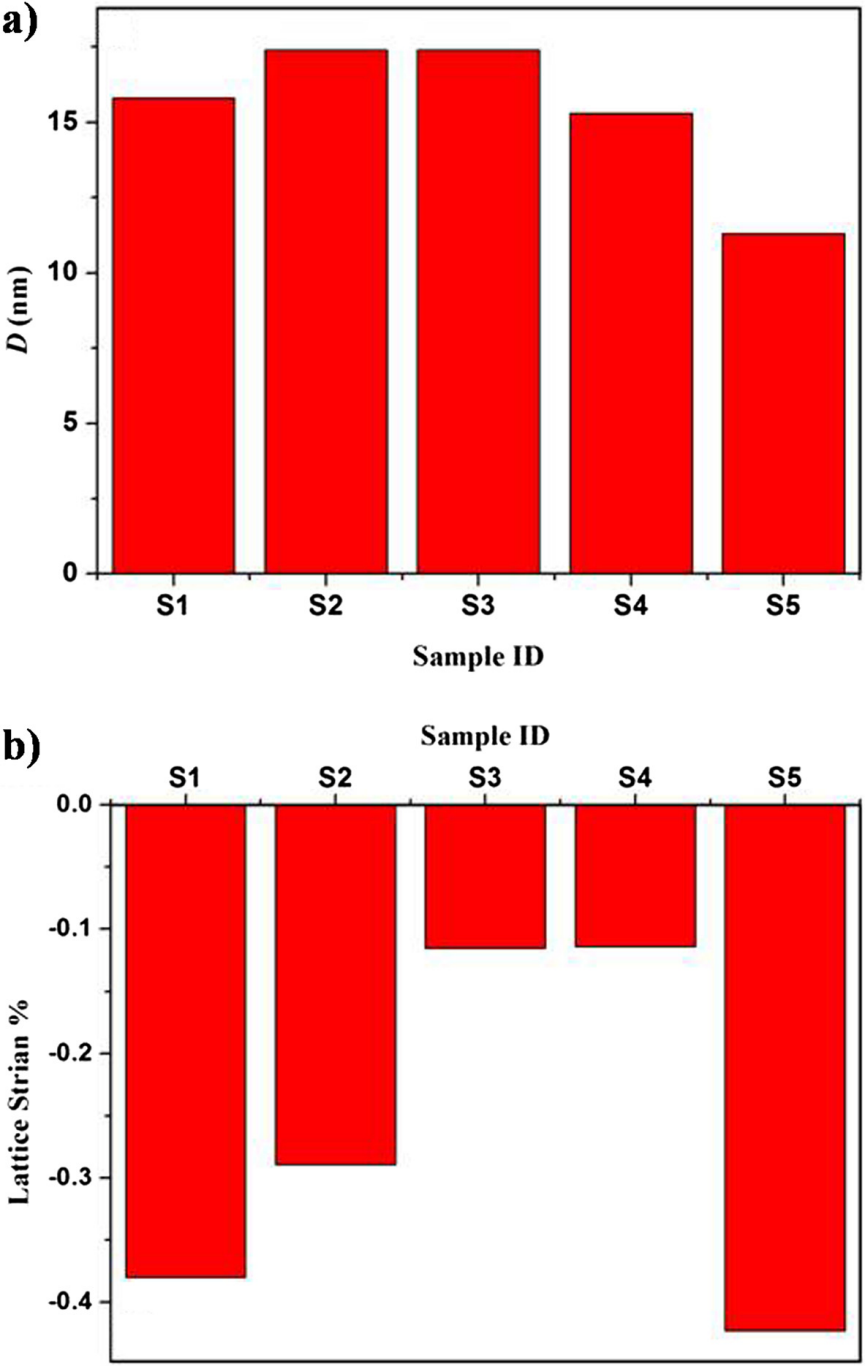
Crystallite size (a) and lattice strain (b) of undoped and Cu-doped ZnO nanorods.

To explore more details about the influence of Cu precursors and the concentration on the crystal structure of the grown nanorods, Scherrer's equation [33] was used to estimate the crystallite size (*D*) of the nanorods along the (002) peak. From Figure 2a, the nanorods doped with 1 and 2 at.% from Cu(CH_3_COO)_2_ (S2 and S3, respectively) showed higher crystallite size (*D* = 17.4 nm) compared to the undoped nanorod (S1) (*D* = 15.8 nm). When we use Cu(NO_3_)_2_ as the Cu precursor instead of Cu(CH_3_COO)_2_, the crystallite size decreases from 15.8 nm (for the undoped nanorods) to 11.3 nm (for sample S5). Clearly, the nanorods doped using Cu(NO_3_)_2_ (S4 and S5) had slightly smaller crystallite sizes relative to the ZnO nanorods doped using Cu(CH_3_COO)_2_ (S2 and S3). Such variations in the crystallite size might be the result of the changes in the host lattice parameters due to Cu incorporation [16,27]. The lattice strain of the undoped ZnO nanorods and the Cu-doped ZnO nanorods was calculated using Equation 1.

(1) $$\epsilon_{z}=\frac{c-c_{{}^{\circ}}}{c_{{}^{\circ}}}\times 100,$$

where *c* is the lattice constant (Table 1) of the ZnO nanorods calculated from the XRD measurements, and *c*_*°*_ = 5.206 Å is the lattice constant of the standard unstrained ZnO. From Figure 2b, all samples showed a compressive strain. It appears that when Cu(CH_3_COO)_2_ is used as the Cu precursor, the lattice strain decreases with the increase in the Cu concentration, reaching its minimum (−0.115%) for the nanorods doped with 2 at.% (sample S3). On the contrary, when Cu(NO_3_)_2_ is used instead of Cu(CH_3_COO)_2_, the lattice strain decreased significantly (−0.114%) for 1 at.% Cu (S4) and increased to maximum when 2 at.% is added (sample S5). It is evident that sample S4 (doped with 1 at.% from Cu(NO_3_)_2_) showed a minimum lattice strain (Figure 2b). This result suggests that the Cu dopants in sample S4 took proper sites in the ZnO lattice. Generally, the substitution of Zn^2+^ by Cu^2+^ would lead to a change in the lattice parameters [18,27]. However, the pronounced changes in the lattice strain when Cu(NO_3_)_2_ is used as the Cu precursor (samples S4 and S5) suggest that the concentration of OH^−^ in the aqueous solution plays an important role in the crystalline quality of the grown nanorods.

### Morphology

**Figure 3:**
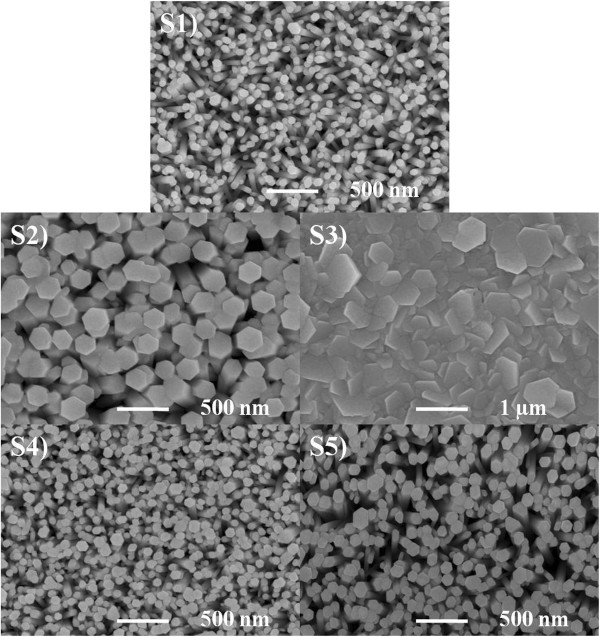
SEM images of the undoped and Cu-doped ZnO nanorods.

The morphology of the nanorods was investigated by scanning electron microscopy. The top-view SEM images for the undoped and Cu-doped ZnO nanorods are shown in Figure 3. The density and diameters of the nanorods showed dependency on Cu precursor and concentration. It can be seen that the average rod diameter increases from approximately 75 nm for undoped nanorods (sample S1) to approximately 210 nm when 1 at.% Cu is added from Cu(CH_3_COO)_2_ (sample S2),while when 2 at.% (sample S3) is added from the same precursor, the nanorods aggregated and the structure becomes compact. On the other hand, when 1 at.% of Cu (sample S4) is added from Cu(NO_3_)_2_, the average nanorod diameter increases slightly relative to the undoped nanorods. Increasing the Cu content to 2 at.% (sample S5) from Cu(NO_3_)_2_, the average nanorod diameter increases to approximately 120 nm.

**Figure 4:**
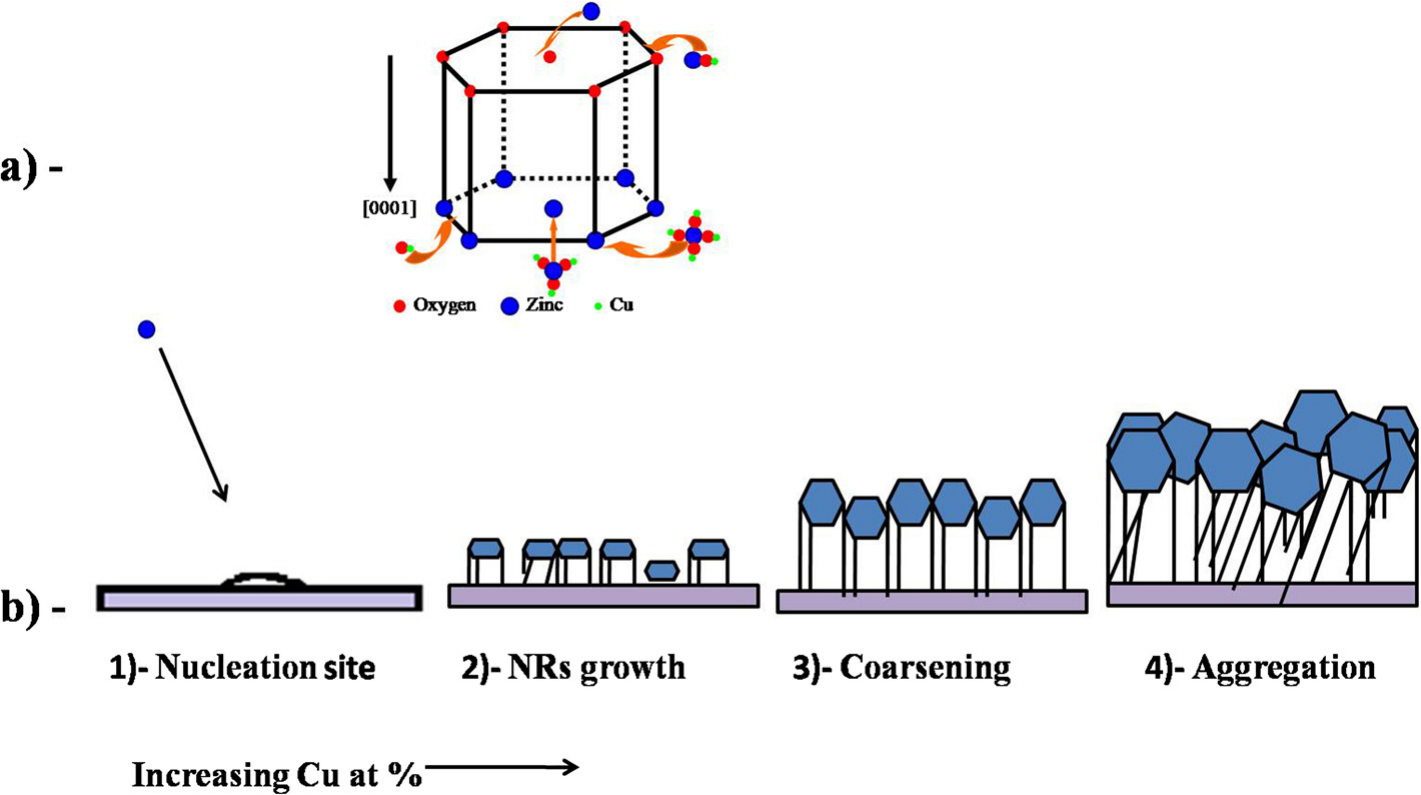
Schematics of ZnO unit cell (a) and nanorod growth and aggregation (b).

The variations in the nanorod diameters and densities as functions of Cu concentration and precursors are explained in Figure 4a,b. The ZnO unit cell is shown in Figure 4a, where the cations (zinc ions) and the anions (oxygen ions) are arranged alternatively along the *c*-axis perpendicular to the substrate. Basically, the nanorod diameter and density are highly affected by the density of the nucleation sites and the pH value of the aqueous solution. Therefore, introducing Cu dopants into the reaction path would increase the nucleation density and hence enhance the growth rate, which in turn, results in a coarsening and lateral aggregation of the nanorods.

The reason why the nanorods doped with Cu(CH_3_COO)_2_ exhibited a larger diameter compared to the nanorods doped with the same concentration of Cu(NO_3_)_2_ is that as shown in Equations 2 and 3, both Cu(CH_3_COO)_2_ and Cu(NO_3_)_2_ release the same concentration of Cu^2+^. Therefore, the anion concentration is a determinant factor.

(2) $$\mathrm{Cu}{\left({\mathrm{CH}}_{3}\mathrm{COO}\right)}_{2}\to {\mathrm{Cu}}^{2+}+{\mathrm{CH}}_{3}{\mathrm{COO}}^{-}$$

(3) $$\mathrm{Cu}{\left({\mathrm{NO}}_{3}\right)}_{2}\to {\mathrm{Cu}}^{2+}+2{\mathrm{NO}}_{3}^{-}$$

The two different anions CH_*3*_COO^*−*^ and ${\mathrm{NO}}_{3}^{-}$ will affect the nanorod growth process in different ways. In the hydrolysis process of CH_*3*_COO^−^, more OH^−^ will be released when the amount of OH^−^ in the aqueous solution decreases (Equation 4). Accordingly, both lateral and vertical growth rates will increase with the increase of Cu(CH_3_COO)_2_.

(4) $${\mathrm{CH}}_{3}{\mathrm{COO}}^{-}+H_{2}O↔{\mathrm{CH}}_{3}\mathrm{COOH}+{\mathrm{OH}}^{-}$$

Conversely, the lack of hydrolysis process in Cu(NO_3_)_2_ would lead to a low concentration of OH^−^, which may slowdown the growth rate [34].

### Photoluminescence

**Figure 5:**
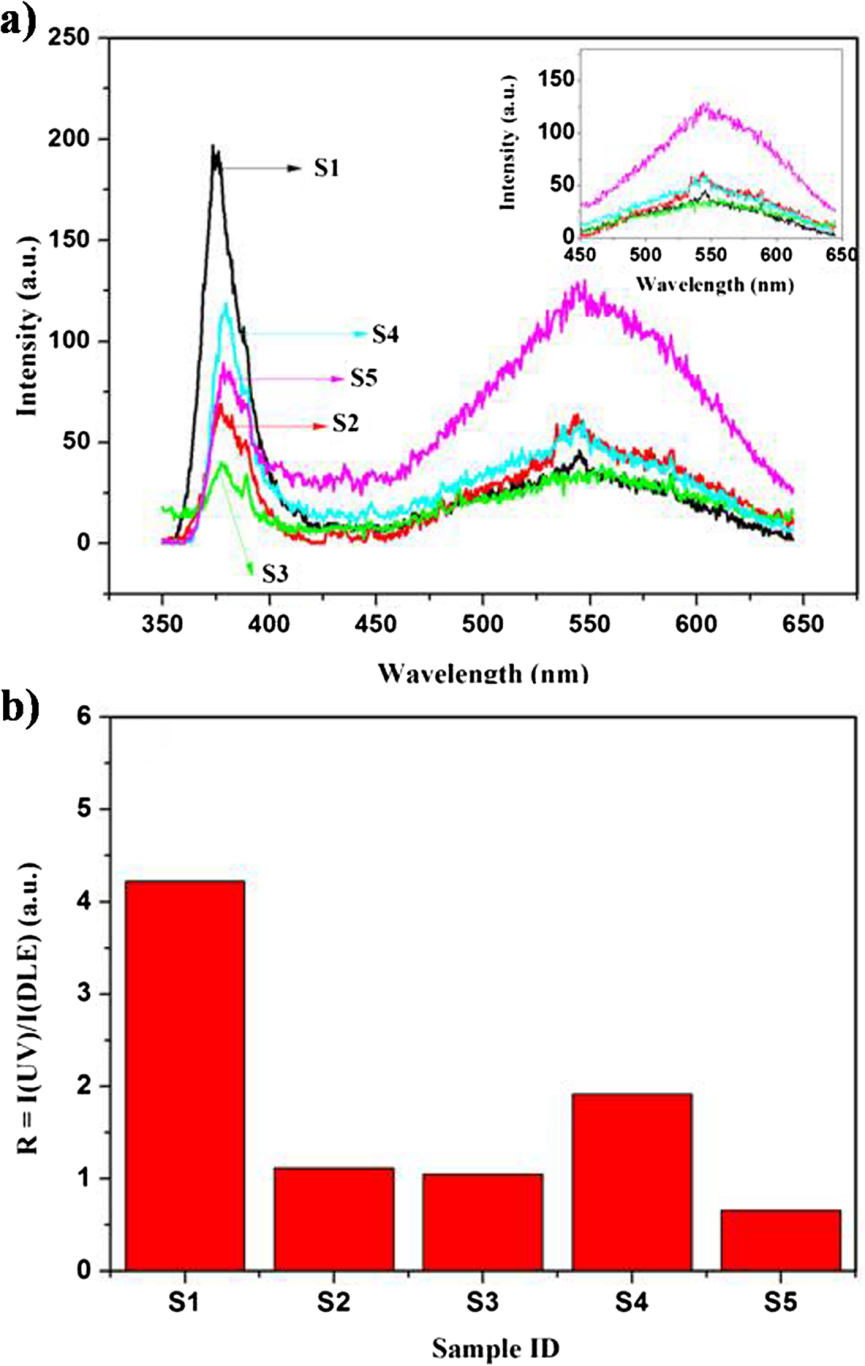
**PL spectra and relative ratio. (a)** Room-temperature PL spectra of undoped and Cu-doped ZnO nanorods; the inset shows the blue-green emission bands. **(b)** The relative ratio of PL intensity (*R* = *I*(UV)/*I*(DLE)).

Room-temperature photoluminescence spectra of all the samples are shown in Figure 5a. All samples exhibited two dominant peaks. The first and sharpest peak is centered on 378 nm and was assigned to the near-band edge (NBE) emission or to the free exciton emission. The intensity of the NBE emission decreases with the increase of Cu concentration for both precursors Cu(CH_3_COO)_2_ and Cu(NO_3_)_2_. This may have resulted from the formation of the nonradiative centers in the Cu-doped samples [28]. In comparison between the two precursors, the nanorods doped with Cu(NO_3_)_2_ (samples S4 and S5) showed a higher NBE emission compared to the nanorods doped with Cu(CH_3_COO)_2_ (samples S2 and S3). This observation could be due to the higher anion concentration in samples S2 and S3 [35]. The UV emission peak of the Cu-doped samples showed a small redshift (approximately 6 nm) relative to the undoped ZnO, where the shift is clearer for the samples doped with Cu(NO_3_) (S4 and S5). This may be attributed to the rigid shift in the valence and the conduction bands due to the coupling of the band electrons and the localized Cu^2+^ impurity spin [16]. It can be observed that there is a small shoulder at around 390 nm, and it becomes pronounced for sample 3, which is doped with 2 at.% Cu from Cu(CH_3_COO)_2_, and this shoulder is ascribed to the free electron-shallow acceptor transitions [25,26]. Additionally, there is a luminescence peak at around 544 nm, which is called the deep-level emission (DLE) or blue-green emission band. When 1 at.% Cu is added from Cu(CH_3_COO)_2_, the intensity of this peak increased slightly (sample S2) and decreased again when 2 at.% Cu is added from the same precursor (sample S3),becoming nearly identical with the undoped ZnO nanorods (sample S1). This result suggests that the green emission is independent of Cu concentration. On the other hand, when we use Cu(NO_3_)_2_ as the Cu source (samples S4 and S5), the green emission enhanced significantly for sample S5 (doped with 2 at.%). Interestingly, the origin of the green emission is questionable because it has been observed in both undoped and Cu-doped ZnO nanorod samples. Vanheusden et al. [36] attributed the green emission to the transitions between the photoexcited holes and singly ionized oxygen vacancies. Based on these arguments, the high oxygen vacancy concentration may be responsible for the higher green emission intensity of sample S5. Additionally, the ratio (*R*) of the NBE emission intensity to the DLE intensity is shown in Figure 5b. The *R* decreases with the increase of Cu concentration.

### Transmittance

**Figure 6:**
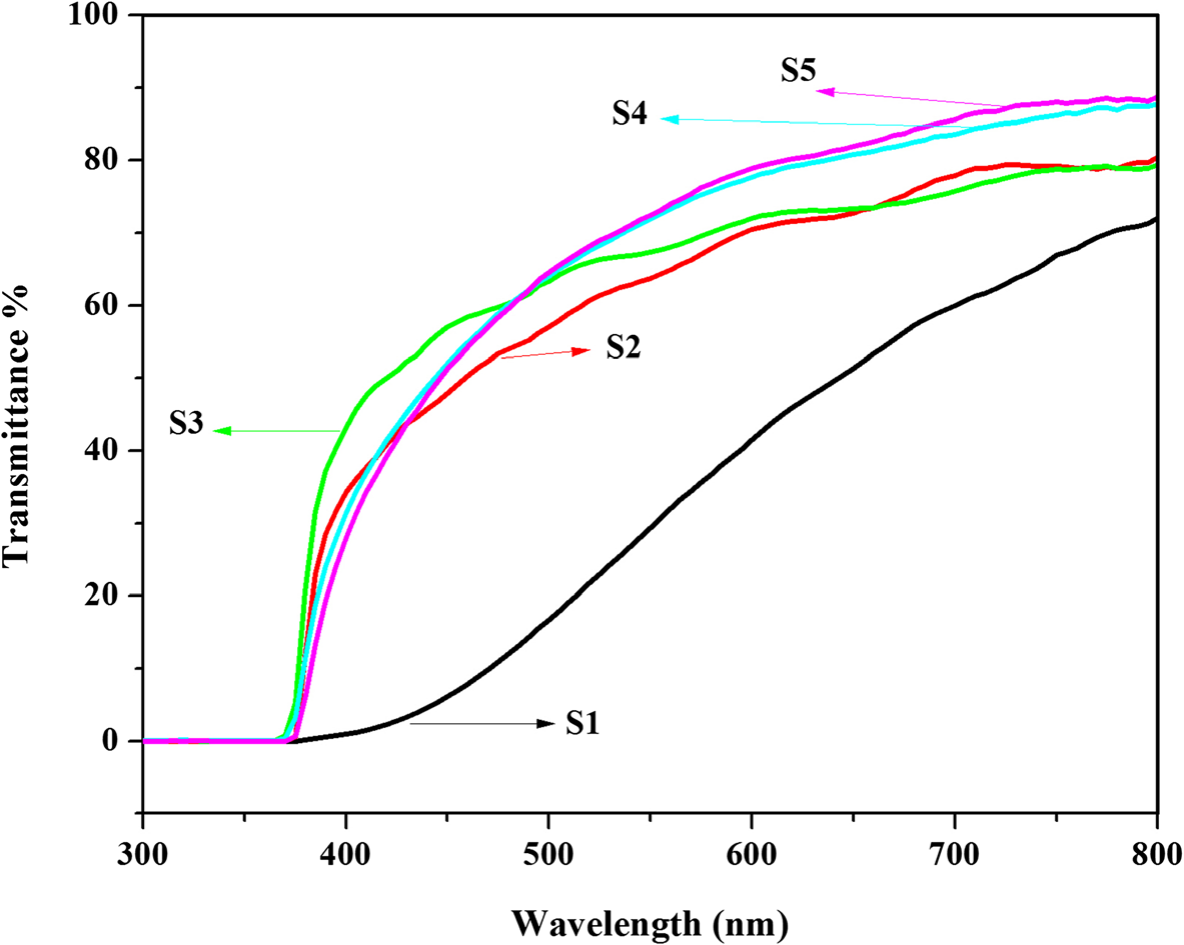
Total transmittance spectra of undoped and the Cu-doped ZnO nanorods.

Figure 6 shows the total transmittance spectra for the undoped and Cu-doped ZnO nanorods, where all samples are found to be transparent in the visible region. It is evident that the rise of the absorption edge near the band edge for the pure ZnO nanorods (sample S1) increased gradually, while it becomes sharper for the Cu-doped ZnO nanorods (samples S2 to S5), indicating the presence of localized states within the bandgap. The undoped ZnO nanorods (sample S1) showed lower transmittance (approximately 70%) compared to the Cu-doped ZnO nanorods. This could be attributed to the scattering either from the unfilled inter-columnar volume and voids or from the inclined nanorods. Using Cu(CH_3_COO)_2_ as the Cu source (samples S2 and S3), the total transmittance increased, reaching approximately 80%, and was found to be independent on the amount of Cu dopants. Comparatively, using Cu(NO_3_)_2_ as the Cu precursor (samples S4 and S5), the total transmittance increased further, reaching approximately 90%. Lin et al. [37] related the presence of oxygen vacancies to the transmittance ratio, where lower transmittance indicates that there are more oxygen vacancies and vice versa. However, in the study reported here, we can attribute the reduction in the total transmittance to the increase in the rod diameter for the samples doped with Cu(CH_3_COO)_2_. It can be seen that at the absorption edge for Cu-doped ZnO nanorods, the slight blueshift indicates that the bandgap was tuned by the incorporation of the Cu dopants. It may be observed that there are obvious interference fluctuations in the transmission spectra when Cu(CH_3_COO)_2_ was used as the Cu precursor (samples S2 and S3). These fluctuations can be attributed to the presence of scattering centers [36].

## Conclusions

In conclusion, we explored the effect of Cu precursors (Cu(CH_3_COO)_2_ and Cu(NO_3_)_2_) and concentration on the structural, morphological, and optical properties of the hydrothermally synthesized Cu-doped ZnO nanorods. The XRD results revealed that the slight changes in the lattice parameters have occurred due to the substitution of Zn^2+^ by Cu^2+^ and the formation of defect complexes. The nanorods doped with Cu(NO_3_)_2_ had less crystallinity than the nanorods doped with Cu(CH_3_COO)_2_, where the maximum compressive lattice strain (−0.423%) was obtained when 2 at.% of Cu was added from Cu(NO_3_)_2_. From the SEM studies, Cu(CH_3_COO)_2_ was found to be an effective precursor for the formation of Cu-doped ZnO nanorods with large diameter. Conversely, Cu-doped ZnO nanorods with a small diameter (approximately 120 nm when 2 at.% was added) can be obtained when Cu(NO_3_)_2_ is used as a Cu precursor due to the lack of hydrolysis process. UV and green emission peaks at 378 and 544 nm were observed for all samples and are attributed to the near-band edge UV emission and the defect-related emission, respectively. A redshift of approximately 6 nm in the UV emission band was seen for the Cu-doped ZnO nanorods and was attributed to the rigid shift in the valence and the conduction bands due to the coupling of the band electrons and the localized Cu^2+^ impurity spin. Irrespective of Cu concentration, the nanorods doped with Cu(CH_3_COO)_2_ showed a transmittance of approximately 80% in the visible range, while the nanorods doped with Cu(NO_3_)_2_ showed a rather high transmittance (approximately 90%). The obtained results are comparable with the previous results. In conclusion, by choosing a suitable Cu precursor and concentration, we can control the diameter of Cu-doped ZnO nanorods, which is important for the fabrication of nano-optoelectronic devices.

## Competing interests

The authors declare that they have no competing interests.

## Authors’ contributions

MB fabricated all the samples, performed the XRD and transmission measurements, and wrote the manuscript. DW performed the PL and FESEM measurements. JW participated in the discussion and manuscript writing. JS and QL contributed in the preparation of some samples. YY, QY, and SJ contributed with valuable discussions. All authors read and approved the final manuscript.

## Authors’ information

MB obtained his MSc degree in nanoscience from Lund University, Sweden. He is currently a Ph.D. student in Harbin Institute of Technology. His research interests include fabrication and properties of metal-doped ZnO nanostructures. DW is an MSc student in Harbin Institute of Technology. His research interests include fabrication and properties of ZnO thin films. JW obtained his Ph.D. degree from Jilin University. He is currently a full professor at Harbin Institute of Technology. His research interests cover pure and doped ZnO nanomaterials, solar cell, and optoelectronic devices. QL is an MSc student at Harbin Institute of Technology. Her research interests include fabrication and properties of p-type ZnO thin films. JS is an MSc student in Harbin Institute of Technology. His research interests include fabrication and properties of ZnO UV detectors. YY obtained his MSc degree in engineering from Harbin Institute of Technology. He is currently a Ph.D. student in Harbin Institute of Technology. His research interests include fabrication and properties of metal oxide solar cells. QY is currently a full professor at Harbin Institute of Technology. His research interests cover metal oxide nanomaterials, solar cell, and gas sensors. SJ is currently a full professor at Harbin Institute of Technology. Her research interests cover pure and doped ZnO nanomaterials.
